# Various Items

**Published:** 1883-01

**Authors:** 


IN the Khoyra district, India, which comprises a considerable portion of the Sunderbunds, more than fifty people—timber cutting and collecting in the jungle—were killed by tigers during the last official year.

THE gradual emancipation of the slaves in Brazil has not only permitted the country to prepare itself for the adoption of free labor, but its influence on immigration has also been most marked. From 1864 to 1872 the average yearly number of immigrants was under 9,000. In 1872 a law was passed in favor of gradual emancipation, and from that date to 1879 the immigrants averaged very nearly 23,000 annually—almost three times the average before the enactment. The next two years showed nearly the same results ; but then a notable increase was observed, and from the 1st of January to the 31st of December, 1881, 39,784 immigrants landed in Brazil.

GREAT is the audacity of the young American. The latest instance of this fact is related by the Troy *Times:* "The four-year-old daughter of a well-known divine of this city is disposed to be dictatorial in a cunning way with her older brothers and sisters. While she was acting the wee tyrant over her brother, the other day, her father decided to rebuke her for the first time, and eloquently set forth to her the kindness of her brother, and her duty to be kind in return. When the exhortation had ended, the little auditor, with tearful eyes. and frame trembling with emotion, strode up to her venerable sire, and striking an attitude, said, between her sobs, 'Y-you use too m-many words.' The father vainly endeavored to suppress his laughter as he went to his study and proceeded to cut down his next Sunday's sermon."

